# Supplementary material for: Impact of the COVID-19 Pandemic on Cancer Diagnosis in Madrid (Spain) Based on the RTMAD Tumor Registry (2019–2021)
Source: Cancers (Basel). 2023 Mar 14;15(6):1753. doi: 10.3390/cancers15061753 (PMC10046347; doi:10.3390/cancers15061753)
Supplement: Supplementary file 1 [file cancers-15-01753-s001.zip › cancers-2108333-supplementary.pdf]

Madrid Cancer Registry (RTMAD) investigators and participating institutions:

Beatriz López Martinez-Bernal. Hospital General Universitario Gregorio Marañón.
Oscar Toldos González. Hospital Universitario Doce Octubre.
Mónica García-Cosio Piqueras. Hospital Universitario Ramón y Cajal.
Ana Cristina García Álvarez. Hospital Universitario La Paz.
Laura Gutiérrez Sainz. Hospital Universitario La Paz.
Cristina Escudero Vela. Hospital Universitario Puerta de Hierro Majadahonda.
Jesús García-Foncillas López. Hospital Universitario Fundación Jiménez Díaz.
Carlos Elvira Martínez. Hospital Universitario Clínico San Carlos.
Rosario Granados Carreño. Hospital Universitario Getafe.
Carmen Salido Campos. Hospital Universitario Príncipe de Asturias.
Ramón Colomer Bosch. Hospital Universitario de La Princesa.
Juan Carlos Cámara Vicario. Hospital Universitario Fundación Alcorcón.
Jesús García-Foncillas López. Hospital Universitario Rey Juan Carlos.
Miguel Ángel López Arenas. Hospital Universitario Infanta Leonor.
Enrique Casado Sáenz. Hospital Infanta Sofía.
María Moreno Díaz. Hospital Universitario de Torrejón.
Montserrat Chao Crecente. Hospital Universitario Severo Ochoa.
Agustín de La Quintana Villegas. Hospital Universitario del Henares.
Ángel Castaño Pascual. Hospital Universitario Fuenlabrada.
Mª Dolores Almagro Cordón. Hospital Universitario de Móstoles.
Juan de Dios Sáez Garrido. Hospital Central de la Defensa.
Jesús García-Foncillas López. Hospital General Universitario Collado Villalba.
Coralia Bueno Muiño. Hospital Universitario Infanta Cristina.
Jesús García-Foncillas López. Hospital Universitario Infanta Elena.
Rafael Carrión Galindo. Hospital del Sureste.
Eugenia Jareño Dorrego. Hospital Universitario Santa Cristina.
Ana Lucia Valencia Mesa. Hospital del Tajo.
Noemi García Miralles. Hospital del Escorial.
Iñigo de Loyola Fente Marco. Hospital Universitario Niño Jesús.
Laura García Acevedo. Hospital de la Cruz Roja.
